# Ambient Ozone Exposure in Czech Forests: A GIS-Based Approach to Spatial Distribution Assessment

**DOI:** 10.1100/2012/123760

**Published:** 2012-04-01

**Authors:** I. Hůnová, J. Horálek, M. Schreiberová, M. Zapletal

**Affiliations:** ^1^Ambient Air Quality Department, Czech Hydrometeorological Institute, 14306 Prague, Czech Republic; ^2^Faculty of Philosophy and Science, Silesian University at Opava, 74601 Opava, Czech Republic; ^3^Ekotoxa s.r.o.-Centre for Environment and Land Assessment, 74601 Opava, Czech Republic

## Abstract

Ambient ozone (O_3_) is an important phytotoxic pollutant, and detailed knowledge of its spatial distribution is becoming increasingly important. The aim of the paper is to compare different spatial interpolation techniques and to recommend the best approach for producing a reliable map for O_3_ with respect to its phytotoxic potential. For evaluation we used real-time ambient O_3_ concentrations measured by UV absorbance from 24 Czech rural sites in the 2007 and 2008 vegetation seasons. We considered eleven approaches for spatial interpolation used for the development of maps for mean vegetation season O_3_ concentrations and the AOT40F exposure index for forests. The uncertainty of maps was assessed by cross-validation analysis. The root mean square error (RMSE) of the map was used as a criterion. Our results indicate that the optimal interpolation approach is linear regression of O_3_ data and altitude with subsequent interpolation of its residuals by ordinary kriging. The relative uncertainty of the map of O_3_ mean for the vegetation season is less than 10%, using the optimal method as for both explored years, and this is a very acceptable value. In the case of AOT40F, however, the relative uncertainty of the map is notably worse, reaching nearly 20% in both examined years.

## 1. Introduction

 Ambient ozone (O_3_) is a widely studied air pollutant [1]. Due to its unsaturated chemical structure, it is highly reactive and contributes to the oxidative power of atmosphere, essential for scavenging many pollutants from the atmosphere [2]. It has important negative impacts on both human health and the environment as acknowledged in numerous studies [3]. Moreover, due to its absorption-radiation abilities, O_3_ is an important greenhouse gas [4, 5], and there are significant interactions between O_3_ and climate change [6]. 

Current ambient O_3 _ levels have increased by approximately two times as compared to those measured over a century ago [7]. Although the magnitude and origin of the hemispheric O_3_ trends are still not completely understood [8], there are indications that background O_3_ levels over the midlatitudes of the Northern Hemisphere have continued to rise over the past three decades within the range of approximately 0.5–2% per year [9]. A significant contribution to O_3_ levels both in Europe and North America originates in East Asia as a result of its dynamic development regarding population growth and increased fossil fuel consumption [10].

For the above reasons, the detailed knowledge of spatial distribution of ambient O_3_ levels is becoming increasingly important. To develop a reliable, accurate, and continuous air pollutant surface predicting the values at locations without measurements is an essential task which we frequently encounter in environmental and health-related studies. This benefit of O_3_ mapping stands out when viewed alongside the increasingly limited financial resources available for costly ambient air quality monitoring networks.

There are a wide range of techniques available for spatial interpolation, the advantages and limitations of which are widely discussed in the scientific literature [11]. In principle these techniques are classified as deterministic (the nearest neighbor and polynomial regression) or stochastic (geostatistical approaches as kriging and cokriging). The difference between these two is that the geostatistical methods use the spatial correlation structure and allow a prediction variability estimate to assess, under certain conditions, prediction accuracy. In between the deterministic and stochastic methods, there are a wide range of radial basis functions or splines. 

The quality of maps of air pollution depends mainly on the quality of the input data measured at the stations, on the number of measuring sites, and also on their spatial distribution [12, 13]. Air pollution measurements, particularly those from online permanent monitoring used in routine monitoring networks, are very costly and so the number of sites is generally very limited. The number of required sites depends obviously on the type of air pollutant and on the representativeness of the measuring site. The representativeness is closely related to proximity of emission sources and topography; more stations are needed in complex terrain in contrast to flat [14]. When developing a surface for pollutants with high spatial variability (due to the importance of their local emission sources), for example, PM, benzo(a)pyrene, or toxic metals, more sites are needed. In contrast, pollutants like O_3_, with a more regional character, depending mostly on regional phenomena such as meteorology and long-range or regional air pollution transport need a less-dense monitoring network. 

Maps of ambient O_3_, in context of its impacts on environment in rural areas, produced by different approaches were published for different regions: United Kingdom [15], Sierra Nevada in California [16–18], and the Carpathians in Europe [19]. Across the EU, mapping of background O_3_ at a fine spatial scale (1 × 1 km) was carried out [20], as well as mapping of exposure index AOT40 at 2 × 2 km grid resolution [21, 22]. Across the Czech Republic, the method of [15] was applied for ozone deposition mapping [23].

In the Czech Republic (CR), ambient O_3_ levels are elevated [24, 25], limit values over vast regions are frequently exceeded, and phytotoxic potential is high [26–28]. The aim of this paper is to compare the different spatial interpolation techniques and to recommend the optimal approach for producing a reliable map of O_3_ with respect to its phytotoxic potential.

## 2. Methods

### 2.1. Ambient Ozone Data

We used real-time O_3_ levels measured at sites in the nationwide air quality monitoring network by UV absorbance, a reference method as declared by the EC [29]. The ozone analyzers used were the Thermo Environmental Instruments (TEI), M49. The samplers were placed ca 2 m above ground. Standard procedures for quality control and quality assurance [29] were applied. We considered O_3 _ seasonal means (April–September) and the exposure index AOT40 for forests—AOT40F [30], calculated according to [31]. The input data were 1 h mean concentrations. Data capture required for calculations of seasonal means was 75%. The AOT40 index as a cumulative variable is very sensitive to the quality of measured data [32, 33] and obviously also to missing values. More stringent requirements are, thus, needed for calculation of AOT40 as compared to the seasonal mean. In cases when we had less than 90% of hourly O_3_ concentrations for the period of April–September for calculating the AOT40F, we corrected it according to [34] so as to prevent underestimation of the O_3_ exposure.

**Figure 1:**
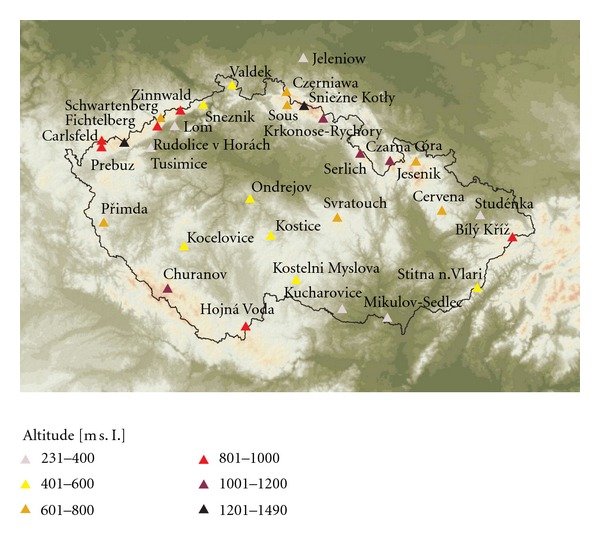
Sites with online monitoring of ambient O_3_ concentrations ranked according to altitude.

With respect to the aim of this study to assess O_3 _ exposure for forests, urban sites were not considered. From a total of 55 sites monitoring real-time O_3_ concentrations across the CR, we accounted only for rural sites as specified by EoI [35]. The rural sites according to EoI are the sites with no important emission sources nearby and which are assumed to be affected only by long-range or regional air pollution transport. Thus, the representativeness of such sites is considerable, mostly in tens to hundreds of kms. The selection of sites resulted in 24 rural sites distributed unevenly across the CR (Figure 1), with more sites at border mountain areas as compared to the interior of the country. Considering that the area of the CR is 79 000 km^2^, the sampling density was approximately one monitor per 3 292 km^2^. 

### 2.2. Spatial Interpolation

Maps for mean vegetation season O_3_ concentrations and for exposure index AOT40F for forests were prepared. For spatial interpolation, we used 24 rural sites run by the CHMI. In addition to the Czech sites, we also used data from selected measuring sites in Germany and Poland to improve the interpolation near border areas (Figure 1). Measuring sites in Slovakia are too distant so they cannot be used. Data from Austrian sites situated near the border were not available. The maps were prepared using ArcGIS Geostatistical Analyst [36] on a grid of 1 × 1 km resolution. A 25 m resolution DEM was used. 

For spatial interpolation, eleven methods were used (Table 1). These methods are adequately referenced in scientific literature, so we comment on them only very briefly. In principle, we use three different interpolation methods using measured data only and two basic methods which combine measured and supplementary data, with four subvariants. 

#### 2.2.1. Interpolation Methods Using Measured Data Only

**(A) Inverse Distance Weighted Method**

The inverse distance weighted method (IDW) is likely to be the most frequently used deterministic method [37]. For interpolation we used

(1) $$\hat{Z}\left(s_{0}\right)=\frac{\sum_{i=1}^{n}Z\left(s_{i}\right)/d_{0i}^{k}}{\sum_{i=1}^{n}1/d_{0i}^{k}},$$

where $\hat{Z}\left(s_{0}\right)$ is the interpolated value of concentration in the point *s*_0_, *Z*(*s*_*i*_) is the measured value of concentration in the *i*th point, i = 1,…, *n*, *d*_0*i*_ is the distance between the interpolated point and the *i*th point with measurement, and *n* is the number of sites used for interpolation.

**(B) Radial Basis Functions Method**

Radial basis functions (RBF) interpolate the measured value while minimizing the total curvature of the surface. The interpolation is described by

(2) $$\hat{Z}\left(s_{0}\right)=\overset{n}{\underset{i=1}{\sum }}w_{i}\cdot \Phi \left(d_{0i}\right)+w_{n+1},$$

where Φ(*x*) is a specific RBF function, *d*_0*i*_ is the distance between the interpolated point and the *i*th point with measurement, *w*_1_, …, *w*_*n*+1_ are the weighting parameters, and *n* is the number of surrounding sites used for interpolation.

Although calculation of the RBF and estimation of its parameters is rather complicated, the computation is simple and fast. The parameters *w*_1_,…, *w*_*n*+1_ are obtained from the system of equations given by

(3) $$\begin{array}{c} \overset{n}{\underset{j=1}{\sum }}w_{j}\Phi \left(d_{ij}\right)+w_{n+1}=Z\left(s_{i}\right),\;i=1,\ldots ,n,\\ \overset{n}{\underset{j=1}{\sum }}w_{j}=-w_{n+1}. \end{array}$$

A more detailed description is given by [36]. Coyle et al., 2002, [15] applied this interpolation technique within his approach for ambient O_3_ mapping for Great Britain.

**(C) Ordinary Kriging**

Ordinary kriging is a geostatistical interpolation method. It considers the statistical model:

(4) $$\begin{array}{c} Z\left(s\right)=\mu +\varepsilon \left(s\right),\;s\in D, \end{array}$$

where *μ* represents the constant mean structure of the concentration field, *ε*(*s*) is a smooth variation plus measurement error (both zero-mean), and *D* is the examined area.

The interpolation is performed according to the equation

(5) $$\begin{array}{c} \hat{Z}\left(s_{0}\right)=\overset{n}{\underset{i=1}{\sum }}\lambda_{i}Z\left(s_{i}\right),\;\;\overset{n}{\underset{i=1}{\sum }}\lambda_{i}=1, \end{array}$$

where $\hat{Z}\left(s_{0}\right)$ is the interpolated value of concentration in the point *s*_0_, *Z*(*s*_*i*_) is the measured value of concentration in the *i*th point, *i* = 1,…, *n*, *n* is the number of surrounding sites used for interpolation, and *λ*_1_, …, *λ*_*n*_ are the weights assumed based on a semivariogram.

The weights **λ*_i_* are derived from a semivariogram **γ**(·) in order to minimize the mean square error. The explicit calculation is achieved by the system of equations given by

(6) $$\begin{array}{c} -\overset{n}{\underset{j=1}{\sum }}\lambda_{j}\gamma \left(s_{i}-s_{j}\right)+\gamma \left(s_{0}-s_{i}\right)-m=0,\;i=1,\ldots ,\;n,\\ \overset{n}{\underset{i=1}{\sum }}\lambda_{i}=1. \end{array}$$

Kriging is a commonly used standard method. For construction of an O_3 _ surface, it was used, for example, by [38–40].

#### 2.2.2. Interpolation Methods Using Both Measured and Auxiliary Data

 The methods described in Section 2.2.1 were used only for the interpolation of the measured O_3 _ concentrations. Apart from these methods, there are others using well-correlated physical relationships between concentrations and other characteristics, for which more complex spatial information is known. The simplest approaches are the linear regression models without spatial interpolation; more complicated are different combinations of linear regression and spatial interpolation.

**(A) Linear Regression Model without Spatial Interpolation**

The basic linear regression model equation considered is

(7) $$\begin{array}{c} Z\left(s\right)=\;c\;+\;a_{1}*X_{1}\left(s\right)+\;a_{2}*X_{2}\left(s\right)+\cdots , \end{array}$$

where *X*_*i*_(*s*) are different supplementary parameters at the point *s*, for *i* = 1, 2,…, *c*,   and  *a*_1_, *a*_2_,…, are the parameters of the linear regression model.

In our case, altitude is used as the auxiliary parameter.

**(B) Linear Regression Model Followed by the Spatial Interpolation of Residuals**

The interpolation is estimated according to

(8) $$\hat{Z}\left(s_{0}\right)=c+a_{1}\cdot X_{1}\left(s_{0}\right)+a_{2}\cdot X_{2}\left(s_{0}\right)+\cdots +\eta \left(s_{0}\right),$$

where $\hat{Z}\left(s_{0}\right)$ is the estimated value of the O_3_ concentration at the point *s*_0_,*X*_1_(*s*_0_), *X*_2_(*s*_0_), …, *X*_*n*_(*s*_0_) are the *n* number of individual auxiliary variables at the point *s*_0_, *c*, *a*_1_, *a*_2_, …, *a*_*n*_ are the *n* selected parameters of the linear regression model calculated at the points of measurement, and *η*(*s*) is the spatial interpolation of the residuals of the linear regression model at the points of measurement.

The output of a dispersion model, altitude, meteorological variables (temperature, relative humidity, global radiation) may be among the auxiliary characteristics. For preparing an O_3_ surface, this method was used, for example, by Loibl et al., 1994 [41], who used relative altitude as the auxiliary characteristics, Horálek et al., 2008 [42], who used model EMEP, altitude, and global radiation as the auxiliary characteristics, and Abraham and Comrie, 2004 [43].

In this study we used the altitude as the sole auxiliary characteristic. The major reason was that preliminary analysis of our data showed the best association between O_3_ concentrations and altitude. Inclusion of meteorological variables did not bring any further benefit to our model. The assumption of linear distribution of O_3_ with altitude was tested prior to the regression analysis.

Different interpolation methods, as described in Section 2.2.1, can be used for interpolation of residuals.

**(C) Interpolation of Mean Afternoon Concentration Minus Regression of Mean Afternoon Increment with Altitude**

Ozone concentrations show diurnal variation. Next to this, mean afternoon increment (i.e., the difference between the mean afternoon concentration and the mean whole-day concentration) is strongly related to altitude; see [15]. Coyle introduced the mapping method in which this regression relation is combined with the spatial interpolation of the afternoon concentration; that is,

(9) $$\hat{Z}\left(s_{0}\right)=\rho \left(s_{0}\right)-R_{\Delta }\left(s_{0}\right),$$

where *ρ*(*s*_0_) is the spatial interpolation of the mean afternoon concentrations at the point *s*_0_ and *R*_Δ_(*s*_0_) is the regression relation of the increment Δ based on altitude at the point *s*_0_.

While Coyle uses minimum curvature function (i.e., one of the RBF function) for the interpolation of afternoon values, we use ordinary kriging, as it shows generally better results; see bellow.

**(D) Interpolation of Mean Afternoon Concentration Minus Regression of Mean Afternoon Increment with Altitude Followed by the Spatial Interpolation of Residuals**

The variant of the method C is the addition of the interpolation of its residuals to the results of this method. As for the method B, different interpolation methods as introduced under Section 2.2.1 are used.

### 2.3. Uncertainty of Maps

We used cross-validation analysis for the assessment of uncertainty of the map. Cross-validation compares a value measured at a monitoring site with an estimated value based on interpolation of values measured at other sites. The root mean square error (RMSE) of the map, calculated according to (10), was used as the uncertainty criterion. RMSE should be as small as possible:

(10) $$\text{RMSE}=\sqrt{\frac{1}{N}\overset{N}{\underset{i=1}{\sum }}{\left(Z\left(s_{i}\right)-\hat{Z}\left(s_{i}\right)\right)}^{2}},$$

where RMSE is the root mean square error of the whole map, *Z*(*s*_*i*_) is the measured concentration at the *i*th site, *i* = 1, …, *N*,$\hat{Z}(s_{i})$ is the concentration at the *i*th site estimated from concentrations measured at other sites, *i* = 1, …, *N*, and *N* is the number of measuring sites.

## 3. Results

The RMSE values comparing the different interpolation techniques both for O_3_ seasonal means and AOT40F are presented in Tables 2 and 3. Considering the average from the vegetation seasons of 2007 and 2008, our results indicate that the optimal interpolation approaches are LR+res_OK and ALR+res_OK. In the case of O_3_ seasonal means, the rankings for both methods were exactly the same, while for AOT40F LR+res_OK slightly outperformed ALR+res_OK. All three interpolation techniques alone—IDW, RBF, and OK—gave much worse results in comparison to linear regression of measured data and altitude with subsequent interpolation of its residuals. This held both for O_3_ seasonal means and AOT40F but was more pronounced for the O_3_ seasonal means.

The relative uncertainty of the map of mean O_3_ for the vegetation season was 8% for LR+res_OK and ALR+res_OK methods for both explored years. This is a thoroughly acceptable value. Even though the IDW method ranking indicated that it was the worst interpolation approach, the relative uncertainty of the map of mean O_3_ for the vegetation season was 13%. In the case of AOT40F, however, the relative uncertainty of the map was notably worse. For LR+res_OK, ranking as the best approach, its relative uncertainty values were 18% in 2007 and 19% in 2008, while for IDW, assessed as the worst approach, its values were 21% for 2007 and 20% for 2008. 

**Figure 2:**
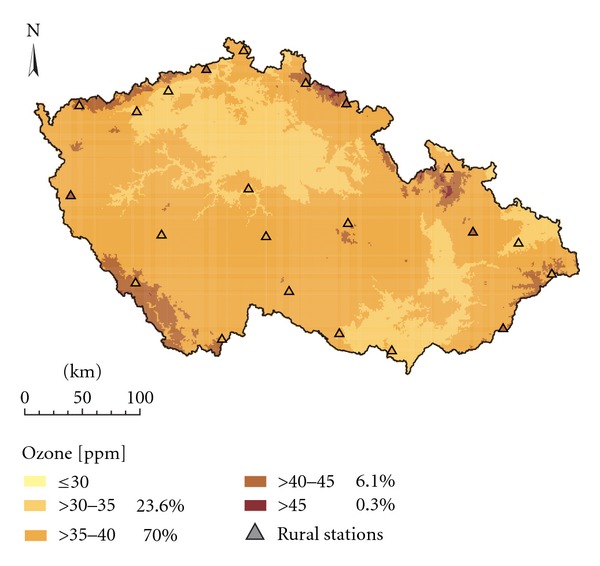
Spatial interpolation of mean O_3_ concentrations in the 2008 vegetation season (ppb), interpolation technique LR + res_OK, grid resolution 1 × 1 km.

**Figure 3:**
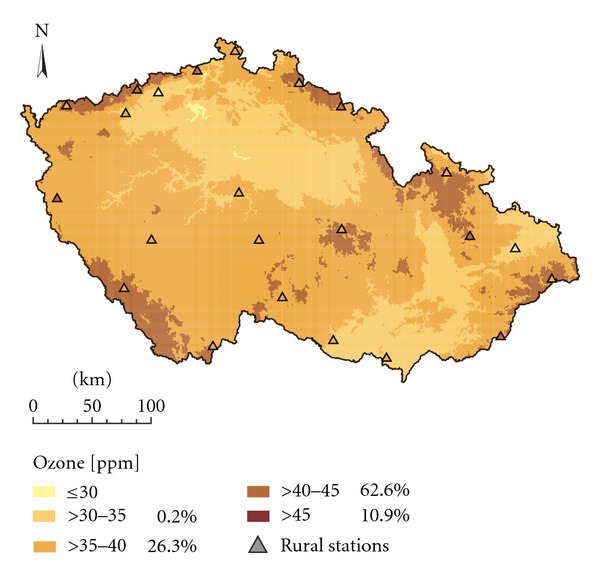
Spatial interpolation of mean O_3_ concentrations in the 2008 vegetation season (ppb), interpolation technique ALR + res_OK, grid resolution 1 × 1 km.

**Figure 4:**
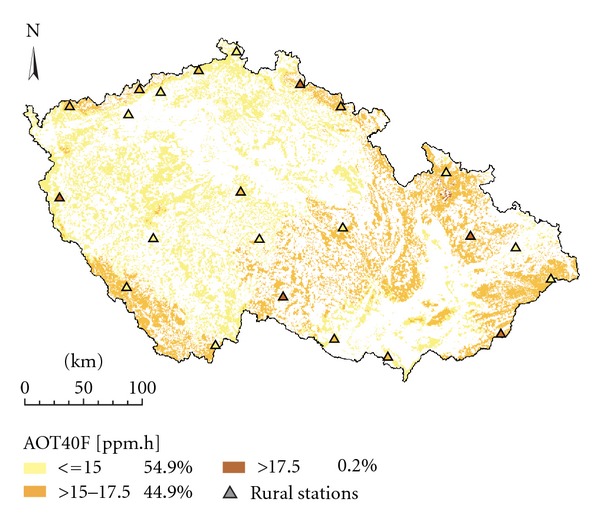
Spatial interpolation of exposure index AOT40F for the 2008 vegetation season (ppb.h), interpolation technique LR + res_OK, grid resolution 1 × 1 km, forested areas.

**Figure 5:**
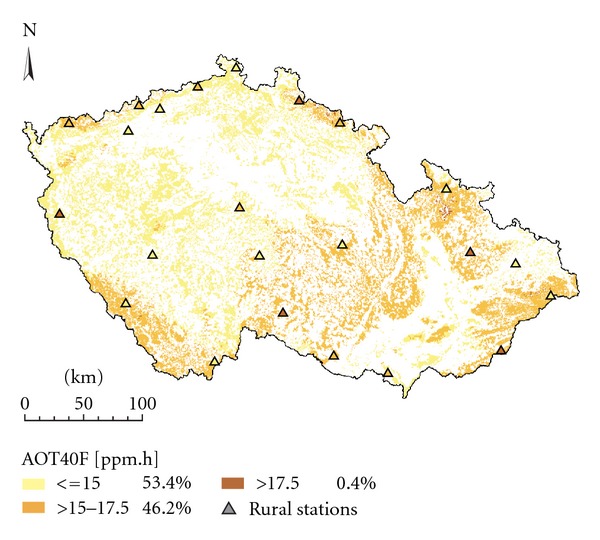
Spatial interpolation of exposure index AOT40F for the 2008 vegetation season (ppb.h), interpolation technique ALR + res_OK, grid resolution 1 × 1 km, forested areas.

**Figure 6:**
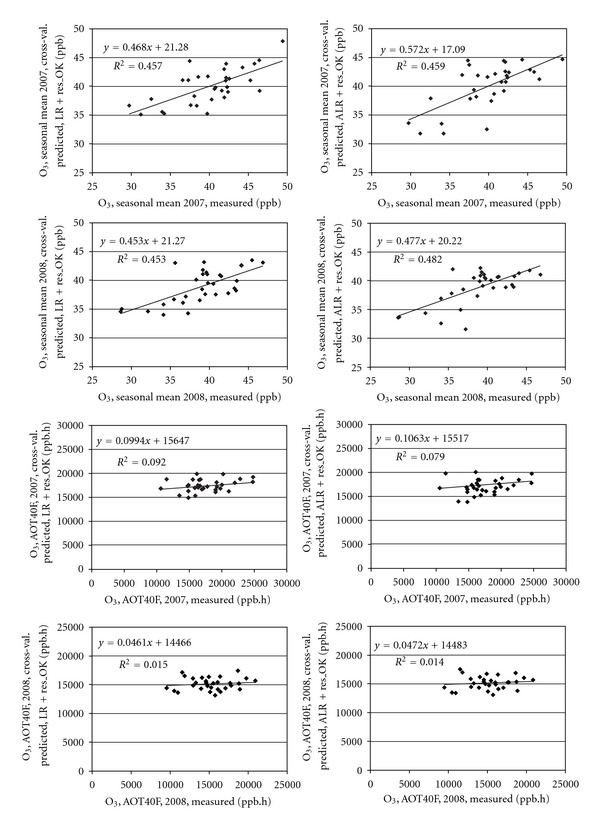
Scatter plots showing the relationship of cross-validation interpolated and measured values for the interpolation techniques LR + res_OK (left) and ALR + res_OK (right) for (a) seasonal mean O_3_ concentrations (b) exposure index AOT40F.

Figures 2 and 3 show the spatial interpolation of mean O_3_ concentrations in the 2008 vegetation season prepared by interpolation techniques LR+res_OK and ALR+res_OK which ranked best in the comparison (Table 2). The two approaches resulted in maps which are very similar and exhibited only minor differences. The same applies for Figures 4 and 5 which show the spatial variability of AOT40F values in the 2008 vegetation season prepared by interpolation techniques LR+res_OK and ALR+res_OK which ranked best in the comparison (Table 3). The relationships between the results of these two interpolation approaches are presented by scatter plots (Figure 6). Notably better results were obtained for O_3 _ seasonal means.

## 4. Discussion

To produce a reliable air pollutant map to predict values in regions without measurements is an essential yet challenging task. Generally, dense monitoring networks are expensive but give a precise picture of spatial variability of a given phenomenon. Sparse sampling and monitoring networks, however, although less expensive, may miss significant spatial features of the studied phenomenon [12]. To make a real monitoring network denser, it is possible, for example, to add virtual measuring sites to improve the quality of interpolation [44]. Currently, however, no rigorous methodology for the determination of the number of monitoring sites sufficient/adequate to develop a reliable air pollutant surface is available [45]. Familiarity with the terrain and various phenomena that could affect the air pollutant concentrations and distributions are among the most important issues [12].

For mapping purposes, a number of techniques are available. There are substantial qualitative differences between the maps derived using different interpolation techniques as shown, for instance, by [46] for the maps of NO_2_. The assessment of performance of the different techniques is extremely important. The maps derived by different interpolation techniques may be compared and evaluated by using the objective criteria, such as cross-validation [42]—when we omit one site in interpolation process and predict its values based on the rest of the sites and then compare the predicted and measured values.

Presented maps are applicable merely for rural areas. The obvious limitation of the maps is the low number of measuring sites which are unevenly distributed across the country. The spatial distribution of sites has strong historic connotations. Originally the measuring sites were located preferentially to more polluted regions, and they still remain in their original setting to observe the long-time trends.

Our results show that using the auxiliary data, in our case the dependence of O_3_ concentrations on altitude in particular, significantly improves the overall quality of the resulting map (see Tables 2 and 3). Meteorology was not factored into the linear regression models as the preliminary analysis of our data showed that including meteorological variables did not bring any further benefit to our model. The likely reason is fairly low number of O_3_ measuring sites. In near future we intend to use the Eulerian photochemical dispersion model CAMx [47] as auxiliary characteristics. The preliminary results seem to be promising. Next to this, it can be stated that the methods using ordinary kriging in its spatial interpolation part show the best results.

If we compare the two methods which ranked as the best, the LR+res_OK (i.e., linear regression of measured values with altitude followed by the interpolation of its residuals using ordinary kriging) approach slightly outperforms ALR+res_OK (i.e., the Coyle's approach [15] followed by the interpolation of its residuals using ordinary kriging) or gives comparable results. ALR+res_OK, however, is much more complicated and demanding for computation and, thus, less practical for application. 

We can reasonably assume that notably worse results of mapping of AOT40F as compared to mean O_3_ concentration for a vegetation season (see Figure 6) are due to more random/incidental factors affecting AOT40. Moreover, exposure index AOT40 is less robust characteristic as compared to mean O_3_ concentrations [33].

Presented maps show the high-resolution O_3_ spatial patterns which can be used for assessment of O_3_ effects on vegetation. Exposure maps in particular are useful for indication of areas with high O_3_ phytotoxic potential for forests and were already used across the Czech forests earlier [26]. Spatial patterns of O_3_ seasonal means are useful for estimation of O_3 _ deposition as published for the Czech coniferous and deciduous forests by [23] and for estimation of stomatal flux.

## 5. Conclusions

We developed reasonable continuous surfaces for ambient O_3_ vegetation season mean concentrations and AOT40F using eleven interpolation approaches. The comparison based on RMSE indicates that linear regression between measured O_3_ data and altitude with subsequent interpolation of its residuals outperforms the interpolation techniques IDW, radial basis functions, and ordinary kriging significantly. This holds for both O_3_ seasonal means and AOT40F, and, in the case of O_3_ seasonal means, this feature is more pronounced as compared to AOT40F. Considering all different aspects, including the results of cross-validation analysis and the demandingness of computation, linear regression of O_3_ data and altitude with subsequent interpolation of its residuals by ordinary kriging can be recommended as the optimal approach out of the eleven spatial interpolation techniques examined. Notably better results in mapping were obtained for mean seasonal O_3_ concentrations in comparison to exposure index AOT40F.

## Figures and Tables

**Table 1:** Methods used for interpolation.

Method	Acronym
Interpolation of measured data by inverse distance weighting	IDW
Interpolation of measured data by radial basis functions	RBF
Interpolation of measured data by ordinary kriging	OK
Linear regression of measured data and altitude	LR
Linear regression of measured data and altitude with subsequent interpolation of its residuals by IDW	LR + res**_**IDW
Linear regression of measured data and altitude with subsequent interpolation of its residuals by RBF	LR + res**_**RBF
Linear regression of measured data and altitude with subsequent interpolation of its residuals by OK	LR + res**_**OK
Interpolation of mean afternoon O_3_ concentration minus regression of mean O_3_ afternoon increment with altitude [15]	ALR
Interpolation of mean afternoon O_3_ concentration minus regression of mean O_3_ afternoon increment with altitude [15] with subsequent interpolation of its residuals by IDW	ALR + res**_**IDW
Interpolation of mean afternoon O_3_ concentration minus regression of mean O_3_ afternoon increment with altitude [15] with subsequent interpolation of its residuals by RBF	ALR + res**_**RBF
Interpolation of mean afternoon O_3_ concentration minus regression of mean O_3_ afternoon increment with altitude [15] altitude with subsequent interpolation of its residuals by OK	ALR + res**_**OK

**Table 2:** RMSE values (ppb) from cross-validation: comparison for different interpolation techniques for O_3_ means, 2007-2008 vegetation seasons.

	Seasonal O_3_ mean (ppb)			
	2007	2008	Average	

	40.15	38.87	39.51	

Interpolation technique	RMSE (ppb)			
	2007	2008	Average	Ranking

IDW	5.23	4.97	5.10	11
RBF	4.92	4.69	4.80	10
OK	4.73	4.67	4.70	9
LR	3.45	3.30	3.38	5
LR + res_IDW	3.49	3.48	3.48	8
LR + res_RBF	3.40	3.37	3.38	6
LR + res_OK	**3.31**	**3.15**	**3.23**	**1**
ALR	3.43	3.38	3.40	7
ALR + res_IDW	3.44	3.22	3.33	4
ALR + res_RBF	3.41	3.15	3.28	3
ALR + res_OK	**3.39**	**3.07**	**3.23**	**1**

**Table 3:** RMSE values (ppb.h) from crossvalidation: comparison for different interpolation techniques for AOT40F, 2007-2008 vegetation seasons.

	AOT40F (ppb.h)			
	2007	2008	Average	

	17570	15202	16386	

Interpolation technique	RMSE (ppb.h)			
	2007	2008	Average	Ranking

IDW	3695	3105	3400	11
RBF	3556	3023	3289	10
OK	3364	2961	3163	9
LR	3438	2841	3140	8
LR + res_IDW	3335	2897	3116	7
LR + res_RBF	3271	2844	3058	6
LR + res_OK	**3169**	**2827**	**2998**	**1**
ALR	3222	2842	3032	3
ALR + res_IDW	3274	2809	3041	4
ALR + res_RBF	3254	2852	3053	5
ALR + res_OK	**3207**	**2850**	**3029**	**2**
